# Host proteins interacting with the Moloney murine leukemia virus integrase: Multiple transcriptional regulators and chromatin binding factors

**DOI:** 10.1186/1742-4690-5-48

**Published:** 2008-06-13

**Authors:** Barbara Studamire, Stephen P Goff

**Affiliations:** 1 Department of Biochemistry and Molecular Biophysics, Columbia University College of Physicians and Surgeons, Hammer Health Sciences Center, Room 1310c, New York 10032, USA; 2 Howard Hughes Medical Institute Columbia University College of Physicians and Surgeons, Hammer Health Sciences Center, Room 1310c, New York 10032, USA; 3 Brooklyn College of CUNY, 2900 Bedford Avenue, Brooklyn, NY 11210, USA

## Abstract

**Background:**

A critical step for retroviral replication is the stable integration of the provirus into the genome of its host. The viral integrase protein is key in this essential step of the retroviral life cycle. Although the basic mechanism of integration by mammalian retroviruses has been well characterized, the factors determining how viral integration events are targeted to particular regions of the genome or to regions of a particular DNA structure remain poorly defined. Significant questions remain regarding the influence of host proteins on the selection of target sites, on the repair of integration intermediates, and on the efficiency of integration.

**Results:**

We describe the results of a yeast two-hybrid screen using Moloney murine leukemia virus integrase as bait to screen murine cDNA libraries for host proteins that interact with the integrase. We identified 27 proteins that interacted with different integrase fusion proteins. The identified proteins include chromatin remodeling, DNA repair and transcription factors (13 proteins); translational regulation factors, helicases, splicing factors and other RNA binding proteins (10 proteins); and transporters or miscellaneous factors (4 proteins). We confirmed the interaction of these proteins with integrase by testing them in the context of other yeast strains with GAL4-DNA binding domain-integrase fusions, and by in vitro binding assays between recombinant proteins. Subsequent analyses revealed that a number of the proteins identified as Mo-MLV integrase interactors also interact with HIV-1 integrase both in yeast and in vitro.

**Conclusion:**

We identify several proteins interacting directly with both MoMLV and HIV-1 integrases that may be common to the integration reaction pathways of both viruses. Many of the proteins identified in the screen are logical interaction partners for integrase, and the validity of a number of the interactions are supported by other studies. In addition, we observe that some of the proteins have documented interactions with other viruses, raising the intriguing possibility that there may be common host proteins used by different viruses. We undertook this screen to identify host factors that might affect integration target site selection, and find that our screens have generated a wealth of putative interacting proteins that merit further investigation.

## Background

A required step for retroviral gene expression and propagation is the stable integration of the double-stranded DNA viral genome into the genome of their hosts. The viral integrase protein is key in this essential step of the retroviral life cycle [1]. The organization of the various integrase structural domains is conserved from retrotransposons to retroviruses, in that they all possess an N-terminal domain containing a Zinc finger motif, an internal catalytic domain known as the D,D(35)E motif, and a C-terminal region that is far less conserved [2,3]. Following virion entry into the cytoplasm, the viral RNA genome is reverse transcribed to form a linear double-stranded DNA molecule. The viral cDNA and integrase enter the nucleus as a large nucleoprotein complex, termed the preintegration complex (PIC) [4]. For Moloney murine leukemia virus (MoMLV), nuclear entry occurs only in mitotic cells, likely reflecting a requirement for disruption of the nuclear membrane [5]. However, human immunodeficiency virus type 1 (HIV-1) does not require disruption of the nuclear membrane to enter the nucleus, and thus non-dividing cells are equally susceptible to infection [6]. The viral DNA ends are processed by integrase, producing recessed 3' OH termini with a free CA dinucleotide at each end of the long terminal repeat (LTR) [7]. The subsequent steps of integration have been well characterized in vitro: the two free 3'-OH viral DNA ends are used, in a nucleophilic attack on the host DNA, to covalently join the viral and host DNA strands, leaving a gapped intermediate with free 5'-phosphodiester viral DNA ends which presumably are repaired by host enzymes [8,9]. Although the basic mechanism of integration by mammalian retroviruses has been well characterized, the factors determining how viral integration events are targeted to particular regions of the genome or to regions of a particular DNA structure remain poorly defined. Thus, significant questions remain regarding the influence of host proteins on the selection of target sites, on the repair of integration intermediates, and on the efficiency of integration.

Early reports of mammalian and avian retroviral systems suggested that the selection of integration sites might be non-random with respect to the chromatin structure of the DNA target, and perhaps with respect to the primary sequence [10-13]. In addition to the early reports, more recent findings suggest that host cellular proteins are involved in the integration reaction and may also play a role in target site selection, as appear to be the case for yeast retrotransposons Ty1, Ty3 and Ty5. For the gypsy-like retroelement Ty3, in vivo targeting to within one or two nucleotides of tRNA gene transcription start sites is most likely mediated by an interaction with TFIIIB and TFIIIC [14]. As another example, the copia-like element Ty1 frequently integrates within 750-bp of the 5'end of tRNA genes [15], and deletion of the RecQ helicase *SGS1 *results in increased multimerization of the Ty1 genome and the transposition of heterogeneous Ty1 multimers [16]. Mutations in Sir4p that disrupt telomeric silencing result in a loss of targeting of the copia-like element Ty5 to heterochromatic regions of DNA, indicating that targeting is controlled by transcriptional modifiers [17].

Identification and biochemical analysis of host proteins known to interact with retroviral integrase proteins has been limited by the difficulty of manipulating the viral proteins in vitro due to poor solubility and aggregation. However, laboratories using a variety of methods have isolated a growing number of HIV integrase-interacting host factors. Many of these factors have been identified by analyzing the components of the PIC and by yeast two-hybrid screening. Among many other applications, yeast two-hybrid analysis [18] has been used successfully to identify host proteins that interact with Mo-MLV RT protein (eRF1) [19]; HIV-1 Gag protein (Cyclophilins A and B) [20] and HIV-1 IN protein (Ini1). Ini1 was the first identified integrase interacting protein [21]. In early studies, HIV-1 integrase was used as the bait to screen an human cDNA library using the yeast two-hybrid system [21]. This screen resulted in the identification and isolation of the SNF5 homologue integrase interactor 1 (Ini1). In the presence of integrase, Ini1 was found to stimulate the DNA-joining reaction in vitro. More recent reports suggest that Ini1 is incorporated into virions and is required for efficient particle production [22].

Human lens epithelium-derived growth factor (LEDGF), the first host cofactor for HIV-1 integration whose role has been most clearly elucidated, was identified both in a yeast two-hybrid screen (S. Emiliani et al., personal communication), and by its association with exogenously expressed HIV-I IN in cells [23]. Subsequent analysis of this factor has suggested a unique role for LEDGF/p75 in nuclear targeting of integrase in HIV-1 infected cells [23,24] and an essential role for LEDGF/p75 in HIV-1 integration [25] and in viral replication [26]. Thus, LEDGF/p75 appears to play a major role in HIV-1 integration and is the first host protein conclusively identified as having an integral and direct role in targeting integration [27].

There have been no reported yeast two-hybrid screens using Mo-MLV integrase as bait, and there are no proteins known to interact directly with MoMLV IN. In an effort to identify host proteins that interact with MoMLV integrase, we performed a series of yeast two-hybrid screens of murine cDNA libraries. Three primary screens were performed which produced 121 putative interacting proteins. We chose to further characterize the interactions of 27 of these factors with MoMLV integrase and to test their interactions with HIV integrase. A subset of the proteins identified was found to interact with HIV-1 integrase. As presented below, we identified three groups of interacting proteins in the screens: Group I, transcription factors and chromatin binding proteins; Group II, RNA binding proteins; and Group III, miscellaneous proteins. A subset of the proteins identified in the screens was tested for binding to recombinant IN proteins in vitro, and by secondary analysis of two-hybrid interactions in different yeast strains. A smaller subset of the proteins identified in the screens was tested with integrase deletions in yeast-two hybrid assays to localize the region of interaction with MoMLV integrase. In this paper, we present the first examples of proteins interacting directly with both MoMLV and HIV-1 integrase in vitro and in vivo in yeast cells. These proteins represent a rich source of candidate interactors that may impact retroviral integration target site selection.

## Results

### Analysis of MoMLV integrase-integrase interactions in the yeast two-hybrid system

**Figure 1:**
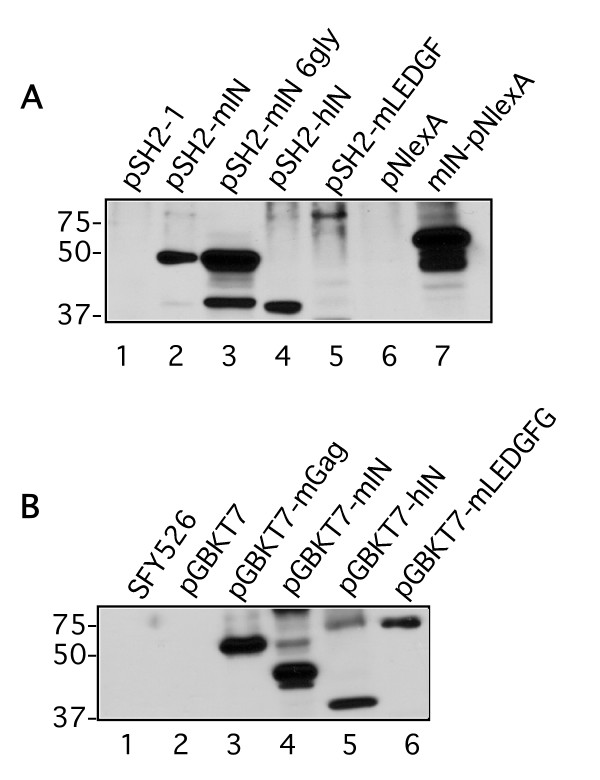
**Expression of DNA binding domain-IN plasmids and control plasmids used in the yeast two-hybrid screens**. **(A) **Lysates from strain CTY10-5d were electrophoresed on 10% SDS-PAGE gels, transferred to PVDF membrane and probed with anti-lexA. Lane 1, pSH2-1 empty vector; lane 2, pSH2-MoMLV IN; lane 3, pSH2-MoMLV IN with 5'six-glycine linker; lane 4, pSH2-HIV-1 IN; lane 5, pSH2-mouse LEDGF; lane 6, pNlexA empty vector; lane 7, MoMLV IN-pNlexA. **(B) **Lysates from strain SFY526 were electrophoresed on 10% SDS-PAGE gels, transferred to PVDF and probed with anti-GAL4-DB. Lane 1, strain without vector; lane 2, pGBKT7 empty vector; lane 3, pGBKT7-MLV Gag; lane 4, pGBKT7-MoMLV IN; lane 5, pGBKT7-HIV-1 IN; lane 6, pGBKT7-mLEDGF.

Lysates from the CTY10-5d yeast strain bearing lexA MLV integrase (pSH2-1 and pNlexA) constructs were examined for protein expression on Western blots probed with an anti-LexA antibody (Figure 1A). To examine potential autonomous activation of the DNA binding domain fusions and to confirm the expected multimerization of MoMLV IN, plasmids pSH2-mIN, pSH2-mIN 6G, and mIN-pNlexA were introduced into the reporter strain CTY10-5d alone, or co-transformed with the GAL4-AD plasmids pGADNOT, pGADNOT-mIN, plasmid pACT2, or pACT2-mIN. Colonies were lifted onto nitrocellulose membranes and stained with X-gal to score for β-galactosidase activity. No self-activation was observed with the two lexA-DB empty vectors, with the lexA-DB-mIN fusions transformed singly, nor with either of the empty GAL4 AD vectors pGADNOT or pACT2 (Table 1 and data not shown). Activation of the β-galactosidase reporter was observed when mIN was expressed in the following plasmid combinations in pair-wise homodimerization tests: pSH2-mIN/pGADNOT-mIN, pSH2-mIN6G/pGADNOT-mIN, pSH2-mIN/pACT2-mIN, pNlexA-mIN/pGADNOT-mIN, and pNlexA-mIN/pACT2-mIN (data not shown). Thus, we were assured that the proposed full-length integrase bait plasmid constructs to be used for the screens and retest assays were appropriately capable of multimerization in vivo, and would produce no background activation of the lexA operator-β-galactosidase reporter fusion.

**Table 1:** Yeast two-hybrid clone interactions with lexA C-terminal and N-terminal fused MoMLV integrase and with C-terminal fused HIV-1 integrase

	**lexA fusions**					**No. isolates in each library**		**Total number isolates**
			
**GALAD fusions**	**pSH2-1**	**pSH2-MLV IN**	**pSH2-HIV IN**	**pNlexA**	**MLV IN-pNlexA**	**WEHI-3B**	**T-cell**	
**Controls**								
pGADNOT	**-**	**-**	**-**	**-**	**-**	na	na	na
pACT2	**-**	**-**	**-**	**-**	**-**	na	na	na
mLEDGF	**-**	**-**	**++**	**nt**	**nt**	na	na	na
HIV-RTp51	**-**	**-**	**+/-**	**-**	**nt**	na	na	na
HIV IN	**-**	**-**	**+++**	**nt**	**nt**	na	na	na
**Gal4-AD clones isolated**								
Fen-1	**-**	**+**	**++**	**-**	**+**	from Fv-1 screen	na	1
Enx-1	**-**	**+**	**+**	**-**	**-**	4	0	4
TFIIE-β subunit	**-**	**+**	**+**	**-**	**+**	3	1	4
Ku70	**+**	**++**	**+++**	**+/-**	**+++**	0	1	1
TBP ABT1	**-**	**++++**	**+**	**-**	**+**	0	2	2
PRC	**-**	**+++**	**++**	**-**	**++**	2	1	3
B-ATF	**-**	**+++**	**+/-**	**-**	**+**	1	0	1
Brd2	**-**	**++++**	**+**	**-**	**+++**	7	2	9
AF9/Mllt3	**-**	**++++**	**+**	**-**	**++**	4	0	4
Baz2b	**-**	**++++**	**+**	**-**	**+++**	1	0	1
Ankrd49	**-**	**++**	**+**	**-**	**-**	1	0	1
Zn finger p15	**-**	**++**	**+/-**	**-**	**+/-**	1	0	1
Zn finger p38	**-**	**+**	**+++**	**-**	**+/-**	1	0	1
SLU7	**-**	**+**	**++**	**-**	**+**	0	1	1
HSL bp	**-**	**++**	**+**	**-**	**++**	0	3	3
TIF3/eIFs2/TRIP1	**-**	**++**	**-**	**-**	**-**	3	0	3
SF3b2	**-**	**+++**	**+++**	**-**	**+++**	4	0	4
SF3a3	**-**	**+++**	**++**	**-**	**++++**	0	1	1
U2Af^26^	**+/-**	**+++**	**+**	**-**	**++**	0	1	1
U5snRNP	**-**	**+**	**+/-**	**-**	**-**	1	0	1
SMN	**-**	**+++**	**+++**	**-**	**+++**	0	1	1
Ddx p18	**-**	**+/-**	**++**	**-**	**+++**	5	0	5
Ddx p68	**-**	**+/-**	**+**	**-**	**+++**	2	0	2
Kif3A	**-**	**+**	**++**	**-**	**+**	2	0	2
Radixin	**+/-**	**+++**	**++**	**-**	**++**	0	1	1
Ran bp 10	**-**	**+**	**++**	**-**	**+**	0	1	1
Trpc2	**+/-**	**+**	**+**	**-**	**+++**	0	1	1

Interactions between MoMLV IN, HIV-1 IN, and the clones isolated in the yeast two-hybrid screen. The pACT or pGADNOT plasmids containing the cDNAs isolated from the yeast two-hybrid screens were introduced into strain CTY10-5d bearing either the pSH2-mIN, mIN-pNlexA, or pSH2-hIN plasmids. Qualitative β-galactosidase colony lift assays were performed. No. of isolates in each library: the number of times a clone identified as the indicated insert was retrieved, specific to each library screened. Total number of times an insert corresponding to each protein was retrieved from all screens. Legend: **- **white; **+/- **pale blue; **+ **light blue; **++ **intermediate blue; **+++**, **++++ **dark blue. Additional controls not shown: pSH2-mLEDGF/pGADNOT-hIN, **+++**; pSH2-mIN/pGADNOT-mLEDGF, **-**; pSH2-mLEDGF/pGADNOT-mIN, **-**.

The MoMLV integrase bait plasmids were also tested for interactions with GAL4 AD fusions of HIV-RT p51 [28] as a negative control, and *Mus musculus *LEDGF (pGADNOT-mLEDGF): no interactions were observed between pSH2-mIN with either of these activation domain plasmids in strain CTY10-5d (Table 1). We did not know if HIV-1 IN and mLEDGF would exhibit an interaction in yeast, so we also tested the lexA DB fusions of HIV-1 IN (pSH2-hIN) with pGADNOT-mLEDGF, and pSH2-mLEDGF with pGADNOT-hIN. The hIN and mLEDGF lexA transformants were examined in the X-gal colony lift assay, and protein expression was examined by Western blot (Figure 1A). Positive interactions were observed in CTY10-5d in both cases (Table 1 and data not shown).

### Interactions of cDNA clones with MoMLV IN and with HIV IN in yeast two-hybrid assays

We examined all of the rescued clones in the context of both vectors used to isolate them in the screens (C-terminal and N-terminal mIN fusions) in colony lift assays. Not all clones interacted with the pSH2-mIN and mIN-pNlexA constructs equally, suggesting that the conformation of the integrase fusion has an impact on its ability to bind the putative interacting protein (Enx-1, ABT1, TIF3, B-ATF, AF9, Ankrd49, U5snRNP, Znfp15, Znfp38, Ddx p18, Ddx p68, and Trpc2; see Table 1). A common problem encountered in yeast two-hybrid assays is that of background reporter activation. Because we observed some background binding of Ku70 with both empty vectors (pSH2-1 and pNlexA; Table 1) we tested the putative Ku70 clone for interaction with pSH2-CLIP170 (CAP-GLY domain containing linker protein 1) as a negative control. There was no interaction between Ku70 and this protein (data not shown), suggesting that the background activation we observed between the empty vectors and Ku70 may be due to the intrinsic DNA binding activity of the acidic domain of the protein. In addition to Ku70, three other clones, Radixin, Trpc2 and U2AF^26 ^also exhibited weak background reporter activation in the CTY10-5d colony lift assay in the context of the empty C-terminal lexA DNA binding domain plasmid pSH2-1. To address this issue, we examined these clones in this strain without the DNA binding domain plasmid. None of these proteins were able to activate the reporter in this context (data not shown), suggesting that the background activation observed may be due to the conformation of bait plasmid used. We speculate that because we observed no activation signal with the empty pNlexA plasmid, and each of these clones were isolated with the mIN-pNlexA fusion, the conformation of the truncated lexA reporter in the empty pSH2-1 vector may expose residues not available for interaction in the full length lexA DB, leading to a spurious interaction peculiar to these clones (Table 1).

The proteins isolated represent novel putative interacting partners for MoMLV IN. As there have been no proteins demonstrated conclusively to interact directly with MoMLV IN, and because relatively few HIV-1 IN interacting proteins have been identified, we examined our putative MoMLV IN interactors with HIV-1 IN in yeast two-hybrid assays. Four of the proteins that interacted with mIN interacted equally strongly with hIN. Those that exhibited robust interactions with hIN were Ku70, Znfp38, SF3b2, and SMN, and the interactions between hIN with Ku70 and hIN with Znfp38 were stronger than the interactions observed between mIN and these proteins (Table 1). Intermediate interactions were observed for hIN and Fen-1, PRC, SLU7, SF3a3, Ddx p18, Kif3A, Radixin, and Ran bp10. Some of the proteins isolated in the screen did not interact with hIN at all in these assays (TIF3), or exhibited relatively moderate interactions (Table 1).

### Yeast two-hybrid cDNA library screens

We performed a pilot yeast two-hybrid screen of a mouse WEHI-3B cDNA library in the GAL4 activation domain plasmid pGADNOT using the plasmids pSH2-mIN and pSH2-mIN 6G as baits in strain CTY10-5d. Our pilot screen yielded a high percentage of interacting clones (96 putative interacting clones, data not shown). Due to the large number of interactors isolated in the first screen, we performed two additional independent screens of a mouse T-cell cDNA library in the GAL4 AD plasmid pACT2 in a different isolate of strain CTY10-5d with both C-terminal and an N-terminal fusions of MoMLV integrase as baits. In the T-cell library screen, we obtained 25 interacting clones (see Table S1 in Additional file 1).

We re-examined the phenotypes of each clone identified in the WEHI-3B and T-cell library screens in strain CTY10-5d. We rescued a total of 121 plasmids from yeast and retested each of these putative interacting plasmids with pSH2-mIN and mIN-pNlexA in the X-gal colony lift assay in a minimum of three independent transformations. Of the 121 plasmids rescued, we chose 27 of the clones that retested successfully to characterize on the basis of their phenotypes in the colony lift assay (intensity of activation based on blue color), the number of times the gene was isolated, and our interest in their proposed functions. There are a number of other clones identified in the screens that remain to be examined in greater detail and are not included in this report, but the level of analysis required is extensive and will be included in another report. The clones presented in this report were placed into three general categories according to functions attributed to them after BLAST [29] and database searches. The proteins identified were categorized as follows and are presented in Table 2: Group I, transcription factors and chromatin binding proteins; Group II, RNA binding and splicing factors; and Group III, miscellaneous and transporter proteins. In cases where we obtained multiple isolates of the same protein, very few of the clones were siblings, as the isolated inserts represent different fragments of these proteins (Table 2, column 2). Three of the interacting proteins identified in the WEHI-3B screen were also identified in the T-cell screen: general transcription factor 2E beta subunit [(TFIIE-β), three isolates from the WEHI-3B library and one from the T-cell library]; peroxisome proliferative activated receptor, gamma, coactivator-related 1 [(PRC), two WEHI-3B and one T-cell isolate]; and bromodomain 2 [(Brd2), alternatively known as RING3 and female sterile homeotic related -1, seven WEHI-3B and two T-cell isolates] (Table 2).

**Table 2:** MoMLV integrase interacting proteins identified in the yeast two-hybrid screens

**Insert aliases**	**Complete residues/peptides retrieved**^a^	**Proposed function/properties**^b^	**GenBank accession Nos. **^c^	**Reference**
**Group I, Chromatin binding and transcription factors**				
Enhancer of zeste homolog 1 (Ezh1/Enx-1/Ezh2)	742/31–292; 31–266; 371–615; 371–641	Polycomb group; chromatin structure maintenance and transcriptional regulation; binds ATRX via SET domain	U52951.1	[93]
Transcription factor IIE, beta subunit (TFIIE-β)	292/18–292; 18–228- gap-249–290; 18–233-gap-247–290; 50–292	Subunit of RNA polII holoenzyme; recruits TFIIH to the PolII-TFIIB-TFIID complex	NM_026584	[94]
Ku70/XRCC6	608/1–608	NHEJ, chromosome maintenance, 70 kD subunit with Ku80 subunit of DNA-PKcs	AB010282	[95]
Flap endonuclease-1 (Fen1)	381/143–381	Removes 5' initiator tRNA from Okazaki fragments; DNA repair in NHEJ and V(D)J	AY014962	[96]
Tata binding protein ABT1 (ABT1)	269/20–269 (2)	Associates with Tata binding protein and activates basal transcription of class II promoters	AB021860	[97]
B-Activating transcription factor (B-ATF)	120/1–120	AP-1/ATF superfamily; Basic leucine zipper transcription factor; blocks transformation by H-Ras and v-Fos	AF017021	[48]
Bromodomain containing protein 2 (Brd2)/RING3/female sterile homeotic gene-related 1 (fsrg 1)	798/311–543; 357–541; 530–798; 558–798; 560–798; 562–798; 563–798; 594–798; 595–798	Bromodomain-containing protein; interacts with Latency-associated nuclear antigen (LANA-1) of KHSV; mitogen-activated kinase activity; homolog of Drosophila female sterile homeotic gene	AF045462	[98]
All1 fused translocated to Chromosome 9 (AF9)/mixed lineage-leukemia translocated to 3 (Mllt3)	568/238–428, 476–560; 238–428; 182–362	Pc3 interacting protein; Implicated in H3 hypermethylation; YEATS family member (YNL107w/ENL/'AF-9/and TFIIF small subunit)	AF333960	[39]
Bromodomain adjacent to zinc finger domain, 2B (Baz2b)	2123/615–883	Putative member of ISWI containing chromatin remodeling machinery; DDT, PHD-type zinc finger and putative histone acetyltransferase-Methyl-CpG binding domain (HAT-MBD)	NM_001001182	[47]
Zinc finger p15 (Znfp15)	2192/1526–1808	Binds to Z-box response element between two Pit-1 elements in the growth hormone (GH) promoter; activates GH transcription 100 fold above basal levels	AF017806	[99]
Zinc finger p38 (Znfp38)	555/137–540	Transactivation via SCAN domain; granule cell specification in brain; upregulated in spermatogenesis	NM_011757	[52]
Peroxisome proliferative activated receptor, gamma, coactivator-1 related (PRC)	1644/1181–1644; 1321–1644; 1321–1644	Serum-inducible coactivator of nuclear respiratory factor 1- dependent transcription from RNA pol II promoters; stress response protein	AAH66048	[100]
Ankyrin rep domain 49 (Ankrd49)	238/6–190	Putative transcription factor; contains acidic activation domain; ankyrin repeat domain is similar to SWI6	NM_019683.3	[101]
**Group II, RNA binding proteins**				
Translation initiation factor 3 (TIF3/eIFs2/TRIP1)	325/128–325 (4)	Translation initiation factor; 5 WD repeats; dissociates ribosomes, promotes initiator Met-tRNA and mRNA binding; yeast homolog TUP12 acts as transcriptional repressor	NM_018799	[102]
Splicing factor 3b, subunit 2 (SF3b2)	878/389–844; 385–606; 397–579; 554–781; 397–576	Has putative DNA-binding (bihelical) motif predicted to be involved in chromosomal organization; has SAP domain; proline-rich domain in spliceosome assoc. proteins; basic domain in HLH proteins of MYOD family	NM_030109	[103]
Splicing factor 3a, subunit 3 (SF3a3)	501/318–501	Zinc finger, C2H2-type; RNA splicing, mRNA processing	BC092058	[100]
U2 auxiliary factor 26 (U2AF^26^)	220/53–220	Pre-RNA splicing factor; can replace U2AF^35 ^in vitro	AF419339	[104]
U5 small nuclear ribonucleoprotein (U5 snRNP)	2136/1939–2136	Transcriptional regulation; SNF2 N-terminal domain; conserved C-terminal helicase domain; GTP binding factor; ortholog of *S. cerevisiae *splicing factor Prp8p; mutations in hPRPC8 are autosomal dominants in retinitis pigmentosum	NP_796188	[105]
Step II Splicing factor SLU7	585/27–585	Pre mRNA splicing, required for 3' splice site choice	NM_148673	[106]
Survival motor neuron (SMN)	288/12–254	Component of an import snRNP complex containing GEMIN2, 3, 4, 5, 6 and 7; contains one Tudor domain; deficiency leads to apoptosis	Y12835	[70]
Dead box p18 (Ddx18)	660/366–592; 366–610; 366–660; 366–660; 366–590	RNA-dependent helicase; RNA-dependent ATPase activity; stimulated by ss-RNA	NM_025860.2	[107]
Dead box p68 (Ddx68/Ddx5)	615/247–490; 247–510	RNA-dependent helicase and ATPase activity; stimulated by ss-RNA; interacts with HDAC1	BC129873	[100]
Histone stem loop binding protein (HSLbp)	275/1–275; 1–204; 1–248	RNA transcription events, required for histone pre mRNA processing	NM_009193	[108]
**Group III, Miscellaneous and transport proteins**				
Ran binding protein 10 (Ranbp10)	503/60–387	Interacts with MET (receptor protein tyrosine kinase) via its SPRY domain; does not interact with SOS, competes with Ranbp9 for MET binding; interacts with Ran in vitro	AY337314	[109]
kinesin super family member 3A (Kif3A)	701/443–701; 443–650	Transport of organelles, protein complexes, and mRNAs in a microtubule- and ATP- dependent manner; chromosomal and spindle movements during meiosis and mitosis	NM_008443.2	[110]
Radixin	389/13–330	Member of ezrin, radixin, moesin family of actin binding proteins. Binds directly to ends of actin filaments at plasma membrane	BC053417	[100]
Transient receptor potential prot.2 (TrpC2)	313/3–313	Calcium ion entry channel; putative involvement in DNA damage response	AF111108	[111]

Identities and BLAST search information obtained for MoMLV IN interacting proteins identified in the yeast two-hybrid screens. (^a^) The first number reflects the length of the full-length protein; the next sets of numbers refer to the residues retrieved for each clone. (^b^) Other functions may exist. (^c^) Database accession numbers are current as of May 19, 2007

### Interactions in yeast strain SFY526

In addition to the X-gal colony lift assays in CTY10-5d, we also examined interactions between the integrases and the putative interacting clones in the context of a strain utilizing a GAL4 DNA binding domain-IN fusion protein, and activating a GAL4-responsive reporter. We wished to examine interactions between the integrases and the various GAL4 AD yeast two-hybrid clones in the context of a plasmid with a weak promoter and thus lower expression levels of the fusion bait proteins. Before performing these tests, we subcloned mIN, hIN, MoMLV Gag and mLEDGF into the GAL4 DB plasmid pGBKT7, and examined protein expression in the GAL4 reporter strain SFY526 by Western blotting using an anti-GAL4 DB antibody (Figure 1B). MoMLV Gag/Gag interactions were used as controls in these assays and activation of the GAL4 reporter was observed with cotransformations of pGBKT7-mGag/pACT2-mGag, pGBKT7-mGag/pGADNOT-mGag [30], pGBKT7-hIN/pGADNOT-hIN, pGBKT7-hIN/pGADNOT-mLEDGF, and pGBKT7-mIN/pACT2-mIN (data not shown and Table 3). This series of control assays assured us that there was no integrase-mediated self-activation in this strain. We examined GAL4 DB fusions of mIN and hIN in *S. cerevisiae *strain SFY526 and noted that strong interactions previously observed with both IN proteins were recapitulated in this context for Ku70, Brd2, AF9, Znfp38, Ranbp10, and SMN (Table 3). We also observed that some weaker interactions between hIN and the inserts were not recapitulated for Baz2b, ABT1, SF3a3, and Radixin (data not shown and Table 3).

**Table 3:** Yeast two-hybrid tests in strain SFY526

	**GAL4 DNA binding domain fusions**		
**GAL4 AD fusions**	**pGBKT7**	**pGBKT7-mIN**	**pGBKT7-hIN**
pGADNOT-empty	**-**	**-**	**-**
pACT2-empty	**-**	**-**	**-**
pGADNOT-HIV IN	**-**	**nt**	**++++**
pGADNOT-Gag	**-**	**nt**	**nt**
pGADNOT-mLEDGF	**-**	**-**	**+++**
Fen-1	**-**	**-**	**+**
Enx-1	**-**	**-**	**-**
TFIIE-β	**-**	**-**	**+**
Ku70	**-**	**+**	**++++**
ABT1	**-**	**+**	**-**
B-ATF	**-**	**-**	**-**
BRD2/RING3	**-**	**++++**	**+/-**
AF9/Mllt3	**-**	**+/-**	**+/-**
PRC	**-**	**-**	**+/-**
Baz2b	**-**	**-**	**-**
Zn finger p15	**-**	**-**	**+/-**
Zn finger p38	**-**	**+/-**	**+**
Ankrd49	**-**	**-**	**+/-**
SF3b2	**-**	**-**	**+/-**
SF3a3	**-**	**-**	**-**
U2AF26	**+/-**	**-**	**+/-**
U5snRNP	**+/-**	**-**	**+/-**
splicing factor SLU7	**-**	**-**	**-**
SMN	**-**	**+/-**	**+/-**
Ran bp 10	**+++**	**++++**	**++++**
KIF3A	**-**	**+/-**	**-**
Radixin	**-**	**+**	**-**
Trpc2	**-**	**-**	**+**

Interactions between selected clones isolated in the yeast two-hybrid screens with GAL4-MoMLV IN and GAL4-HIV-IN. The pACT or pGADNOT plasmids containing the cDNAs isolated in the yeast two-hybrid screen were introduced into SFY526 strains bearing the pGBKT7 integrase fusions. Qualitative colony lift assays were performed.

### Deletion analysis of mIN and isolated clones

**Figure 2:**
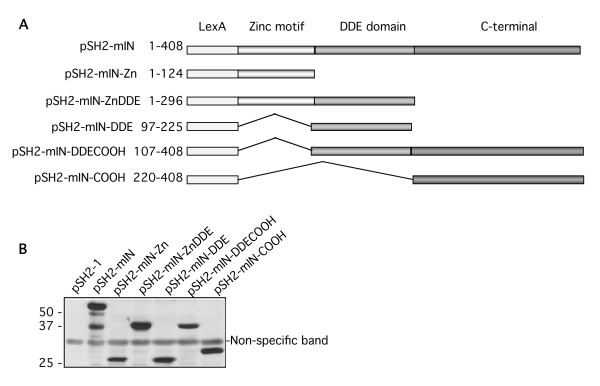
**Construction and expression of MoMLV IN deletion plasmids in CTY10-5d. **(A)**Schematic of pSH2-1 MLV IN truncation constructs.** 1–408, full-length mIN; 1–124, mIN-Zn; 1–296, mIN-ZnDDE; 97–225, mIN-DDE; 107–408, mIN-DDECOOH; 220–408, mIN-COOH. **(B) **Lysates from strain CTY10-5d were electrophoresed on 12% SDS-PAGE gels, transferred to PVDF membranes and probed with anti-LexA. The indicated lysates are shown left to right.

We mapped the region of mIN that interacted with a subset of the clones identified in the yeast two-hybrid screen by introducing deletions into MoMLV IN. We constructed lexA-mIN fusions containing the Zinc binding motif (mIN-Zn), the Zinc binding motif and the catalytic domain (mIN-ZnDDE), the catalytic domain alone (mIN-DDE), the catalytic domain and the C-terminus (mIN-DDECH), and the C-terminus alone (mIN-COOH) (Figure 2A). First, we examined lysates from the mIN deletions to insure that the proteins were expressed (Figure 2B). We then examined the interactions between these deletions and various clones in yeast two-hybrid assays. The most robust interactions were observed between the B-ATF, AF9, Brd2, Enx-1, and ABT1 clones and the mIN-DDECH fusion (Table 4). The interaction between TFIIE-β and the mIN-Zn fusion was stronger than its interaction with any of the other deletion constructs (Table 4). Ku70 interacted with multiple regions, but the most robust interaction was observed between Ku70 and the mIN-Zn fusion (Table 4). These results suggest that there may be discrete regions of mIN that interact with different groups of host factors. More detailed mapping experiments are required to localize the precise residues of mIN responsible for the interactions observed.

**Table 4:** Interactions between pSH2-MoMLV IN deletions and selected yeast two-hybrid interacting proteins

**Fusions**	**lexADB**	**lexA-p66**	**lexA-mIN**	**mIN-Zn**	**mIN-ZnDDE**	**mIN-DDE**	**mIN-DDECH**	**mIN-COOH**
GAL4 AD	**-**	**-**	**-**	**-**	**-**	**-**	**-**	**-**
RT p51	**-**	**++++**	**-**	**nt**	**nt**	**nt**	**nt**	**nt**
mIN	**-**		**++**	**+**	**-**	**+++**	**++**	**-**
B-ATF	**-**		**++**	**-**	**-**	**-**	**+/-**	**-**
AF9	**-**		**++++**	**-**	**-**	**-**	**+++**	**-**
Brd2	**-**		**++**	**-**	**-**	**-**	**+**	**-**
Enx-1	**-**		**+**	**-**	**-**	**-**	**+/-**	**-**
Ku70	**+**		**++**	**+++**	**+**	**++**	**-**	**+/-**
TFIIE-β	**-**		**+**	**+**	**-**	**-**	**-**	**-**
ABT1	**-**		**+++**	**-**	**-**	**-**	**+/-**	**-**

Analyses of MoMLV IN truncations with selected interacting proteins. pSH2-MoMLV IN deletions were introduced into CTY10-5d with the indicated clones in pGADNOT. Qualitative colony lift assays were performed.

### In vitro binding assays

**Figure 3:**
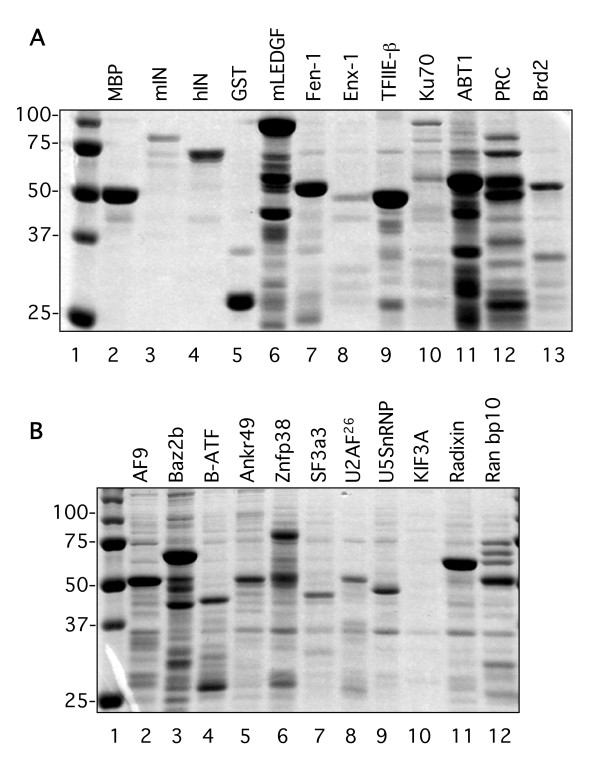
**Expression and binding tests of maltose binding and glutathione-S transferase fusion proteins**. **(A) **MBP lysates were bound to amylose resin, eluted with 15 mM maltose, electrophoresed on 10% SDS-PAGE gels, and stained with Coomassie brilliant blue. Lanes 2–4, expression of pmalc2 (empty vector), pmalc2-mIN, and pmalc2-hIN in TB1 cells. For the GST fusions, the lysates were bound to glutathione sepharose, eluted with 10 mM reduced glutathione, electrophoresed on 10% SDS-PAGE gels and stained with Coomassie brilliant blue. Lanes 5–13, representative loads of GST-yeast two hybrid clones: pGEX2TPL, mLEDGF, Fen-1, Enx-1, TFIIE-β, Ku70, ABT1, PRC, and Brd2. **(B) **Lanes 2–12, GST-yeast-two hybrid clones: AF9, Baz2b, B-ATF, Ankrd49, Znfp38, SF3a3, U2AF^26^, U5snRNP, KIF3A, Radixin, and Ran bp10. Lane 1 in A and B: Molecular weight marker.

**Figure 4:**
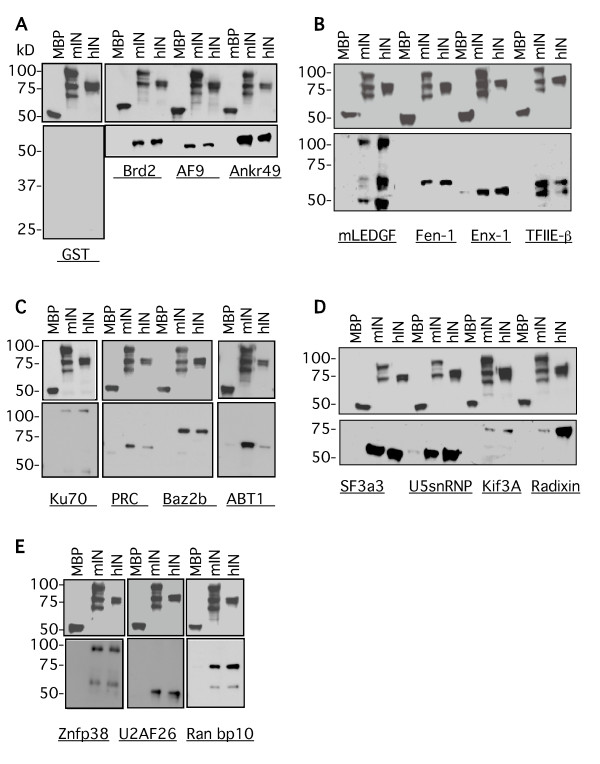
***In vitro*****binding interactions between MoMLV and HIV-1 integrases and selected proteins identified in the yeast two-hybrid screen.** In vitro binding assays between the pmalc2 empty vector (MBP), full-length pmalc2-MoMLV IN (mIN) or full-length pmalc2-HIV-1 IN (hIN) and seventeen of the clones isolated in the screen, plus mLEDGF expressed as GST fusions. The MBP fusion lysates were incubated with amylose resin, washed extensively, resuspended in equal volumes of buffer, and then aliquoted to separate tubes. These tubes were incubated with the GST fusion lysates, washed and eluted with 15 mM maltose. 25 μl of each eluate was electrophoresed on 10 or 12% SDS-PSGE gels, transferred to PVDF membranes, and the same Western was probed with anti-GST, stripped, and then probed with anti-MBP. All Westerns are loaded from left to right: MBP, mIN, and hIN fusion reactions. All upper panels, anti-MBP. All lower panels, anti-GST. **(A) **Maltose binding protein fusions with empty GST vector; MBP fusions with Brd2, AF9, and Ankrd49. **(B) **MBP fusions with mLEDGF, Fen-1, Enx-1, and TFIIE-β. **(C) **MBP fusions with Ku70, PRC, Baz2b, and ABT1. **(D) **MBP fusions with SF3a3, U5snRNP, KIF3A, and Radixin. **(E) **MBP fusions with Znfp38, U2AF^26^, and Ran bp10.

We next examined the interactions between maltose binding protein (MBP)-fused mIN and hIN with 17 of the putative interacting proteins in in vitro binding assays. *E. coli *strains overproducing the MBP IN fusions or the GST fused two-hybrid clones were examined for protein expression (Figure 3A, B). Relative levels of expression were used to determine the amounts of input protein for the binding assays. For the assays, the MBP fusion lysates were first incubated with amylose resin and washed extensively. Lysates from *E. coli *strains overproducing the GST fused two-hybrid subclones were incubated with the washed MBP-amylose resin-bound integrase proteins. We performed these binding assays to determine if the GST proteins could interact specifically with the MBP-integrase fusions. The MBP-IN/GST-putative interacting protein complexes were eluted from the amylose resin by competition with maltose. This was done to resolve *bona fide *complexes between the integrases and the putative interacting fusions, rather than non-specific interactions between the resin and input proteins. There was some C-terminal proteolytic cleavage of both MLV and HIV integrases in these expression studies, the extent of which varied from preparation to preparation, as can be seen by the cleavage products visible in both the Coomassie stained gels and in the Western blots employing these proteins (Figure 3A, lanes 3 and 4 and Figures 4A, B, C, D, and 4E).

In general, the intensity of the interactions between the GST subclones and the two retroviral integrases correlated well with the strength of the interactions observed in the yeast two-hybrid assays. The MBP-mIN fusion interacted with the 17 proteins examined as GST fusions: Brd2, AF9, Ankrd49, Fen-1, Enx-1, TFIIE-β, Ku70, PRC, Baz2b, ABT1, SF3a3, U5snRNP, Kif3A, Radixin, Znfp38, U2AF^26^, and Ranbp10 (Figures 4A, B, C, D, and 4E). The MBP-hIN fusion interacted with 15 of the GST fusions analyzed: Brd2, AF9, Ankrd49, Fen-1, Enx-1, TFIIE-β, Ku70, Baz2b, SF3a3, U5snRNP, Kif3A, Radixin, Znfp38, U2AF^26^, and Ranbp10 (Figures 4A, B, C, D, and 4E). Only weak interactions were observed in vitro between hIN with PRC and ABT1 (Figure 4C). These data confirm and extend the yeast two-hybrid results, indicating that the interactions are likely direct.

Both mIN and hIN proteins interacted to different extents with Ku70, PRC and ABT1, as was observed in their yeast two-hybrid interactions, but both integrases interacted equally with Baz2b in these assays (compare Figure 4C and Table 1). The mIN and hIN integrases exhibited apparent equivalent interactions in vitro with SF3a3, U5snRNP, and Kif3A, although the intensity of their interactions in vivo was dependent on the LexA fusion (Figure 4D and see Table 1). The in vitro interactions between mIN and hIN with Radixin also did not mirror their in vivo interactions, with hIN exhibiting a stronger interaction than mIN with this protein (Figure 4D and see Table 1). Znfp38, U2AF^26 ^and Ran bp10 interacted equally with both integrases (Figure 4E).

**Figure 5:**
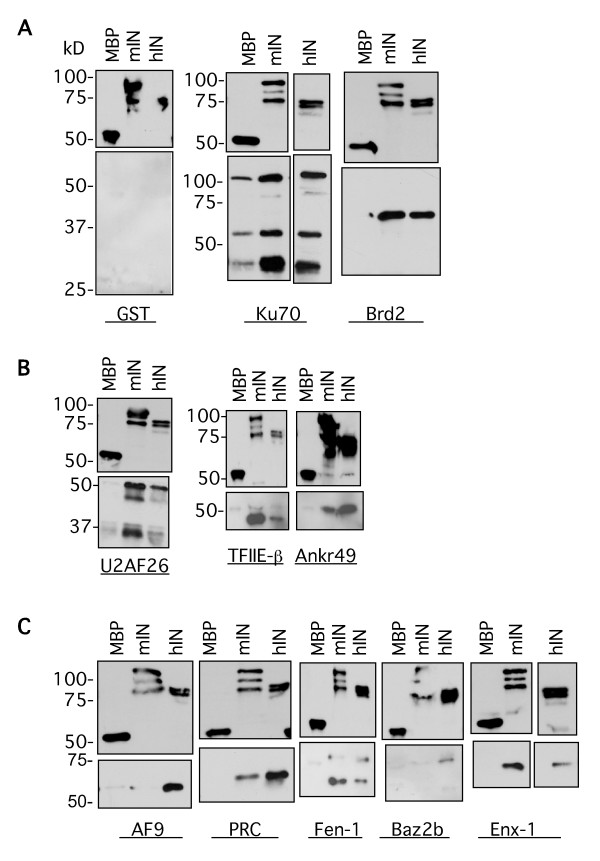
**In vitro binding interactions between MoMLV and HIV-1 integrases and selected proteins after treatment of the lysates with nucleases to eliminate nucleic acid bridges between the proteins**. In vitro binding assays between the empty vector (MBP), full-length pmalc2-MoMLV IN (mIN) or full-length pmalc2-HIV-1 IN (hIN) and a subset of the clones isolated in the screen. All Westerns are loaded from left to right: MBP, mIN, hIN and the indicated GST fusion reactions. Upper panels, anti-MBP. Lower panels, anti-GST. **(A) **Left, maltose binding protein fusions with empty GST vector; right, MBP fusions with Ku70 and Brd2. **(B) **MBP fusions with U2AF^26^, TFIIE-β, and Ankr49. **(C) **MBP fusions with the indicated proteins AF9, PRC, Fen-1, Baz2b, and Enx-1.

The observed in vitro binding of pairs of proteins derived from crude lysates could in principle be facilitated, enhanced, or even mediated entirely by nucleic acids, either RNA or DNA, that bridge the two proteins and mimic direct protein-protein interactions. To address this possibility, a subset of the lysates examined in the pull-down assays were treated with DNase and RNase to eliminate potential contaminating nucleic acids, and the in vitro interaction of the proteins in the lysates was assessed as before. Examination of the lysates for residual nucleic acids showed that the nucleases were highly effective (see Figure S1 in Additional file 2). The binding studies show that the majority of the protein-protein interactions were maintained following nuclease treatment (Figure 5). Of the 18 GST-fusions examined in the in vitro binding assays shown in Figure 4, we examined 13 GST-fusions in assays in which each of the MBP-integrase and GST-clone fusion lysates were treated with DNase and RNase prior to performing the binding reactions. Of the 13 lysates treated, five of the interactions with mIN and hIN were unchanged: Brd2, TFIIE-β, Ankr49, Fen-1 and ABT1 (Figure 5 and data not shown); four were increased, in some cases differentially with respect to the integrase used in the assay: PRC, Ku70, U2AF^26^, and Radixin (Figure 5 and data not shown); and three were decreased: AF9, Baz2b, and mLEDGF (Figure 5 and data not shown). Ten of these binding reactions are shown in Figure 5. No interactions were observed between any of the MBP fusions and the GST vector (Figure 5A, lanes MBP, mIN and hIN). There was some background interaction between Ku70 and MBP, but much lower than the increased interactions observed between this protein with mIN and hIN (compare Figure 5A with Figure 4C). This result may be a function of improved binding between Ku70 and all MBP-fusions due to removal of residual nucleic acids. Of the 14 pairs, the interaction between mIN and U2AF^26 ^(compare Figure 5B with Figure 4E), between AF9 and hIN (compare Figure 5C with Figure 4A), and between PRC and hIN were enhanced (compare Figure 5C with Figure 4C). The interaction between MLV IN with AF9, Baz2b and PRC was decreased in this particular assay, suggesting that some bridging by nucleic acids could not be ruled out (Figure 5C). Binding between Moloney and HIV integrases with Radixin was consistently enhanced following this treatment (data not shown). Although the tests for residual nucleic acids in the lysates suggest that the nuclease treatments were almost completely effective, it is possible that undetected traces of nucleic acids remained, and are still serving as bridges. More extensive testing of the binding interactions following nuclease treatment is required to definitively state that there are no residual nucleic acids remaining in the lysates.

## Discussion

In this report, we used Moloney MLV integrase in the context of two different lexA DNA binding domain fusion vectors as bait to screen two mouse GAL4 activation domain cDNA libraries. We present 27 proteins that interacted with MoMLV integrase in the yeast two-hybrid screens. Twenty of the proteins identified in the screens interact strongly with Mo-MLV IN, and 7 have relatively weaker interactions. We also show that a subset of 12 of these interact strongly with HIV-1 IN, that 11 have intermediate interactions, that three have weak interactions, and that one exhibited no interaction (TIF3). It is of interest to note that the screen has revealed 13 DNA binding proteins, 10 RNA binding proteins, and four proteins involved in transport or signaling. Seven of the isolated clones were examined for their interactions with MLV IN deletions. We found that B-ATF, AF9, Brd2, Enx-1, and ABT1 interacted with the truncated fragment containing both the catalytic and the C-terminal domains. TFIIE-β interacted with the amino terminus of MLV IN and Ku70 interacted with multiple regions of IN. The IN/Ku70 interaction was lost when only the catalytic/C-terminal fragment of IN was expressed. As each of the proteins tested in the truncation assays were DNA binding proteins or transcription factors, we may have identified domains of integrase that interact with a range of transcription factors and DNA binding proteins.

We have examined interactions between 18 of these proteins in vitro using binding assays with both MoMLV and HIV-1 integrases. Of the 18 proteins examined in vitro, we find that 14 exhibited strong interactions with MLV IN and 12 exhibited strong interactions with HIV IN. We find that the intensity of the in vivo interactions in yeast varies between mIN and hIN, which is not surprising, given that the two integrases have little sequence identity and the host protein requirements for their respective integration reaction pathways are presumed to differ, even though the structure of the major functional domains are conserved. Tests for nucleic acid bridging between a subset of the proteins suggest that most of the detected interactions are likely to be direct protein-protein interactions, as also supported by the differential binding of the host proteins to the two integrases.

The results of our assays in yeast and in the in vitro binding assays suggest that there may be many common host proteins used by both viruses. Since the cDNA libraries we screened were murine, we do not presume that all of the clones isolated will exhibit equal effects on both HIV and MLV integration or on virus infectivity, but the isolation of so many putative interacting proteins in our screens merit further investigation for potential roles in the viral life cycle. It is of interest to note that a large group of these proteins, 13 factors, are chromatin binding proteins or transcription factors. Although these various proteins have no obvious simple sequence similarity, it is plausible that the MLV IN protein is recognizing a common feature present on many of these proteins. For example, IN may detect and bind to transcriptional activation domains; the common thread between such proteins may be as inapparent as the acidic protein-protein interaction domains thought to mediate the tethering of transcriptional activators to DNA by promoter or enhancer binding proteins. The significance and consequence of these interactions on viral infectivity and integration await functional analyses.

In early tests for protein-protein binding in vitro, we observed an interaction between HIV-1 IN and LEDGF, a factor widely reported as affecting the efficiency of infection and the target site selection for viral integration. We also observed an unexpected in vitro interaction between mLEDGF and mIN. These proteins did not interact in yeast and there is no documented evidence of an interaction between MLV IN and hLEDGF [31]. When we treated the lysates with nucleases, both the mIN- and hIN-LEDGF interactions disappeared (data not shown), suggesting that the interactions observed in vitro might have only been mediated by nucleic acid bridging. Thus, the significance of the in vitro interaction between mLEDGF and MBP-mIN is unclear. We do not know if the interactions observed between mLEDGF and hIN suggest that mLEDGF could play a similar role in the integration of HIV in mouse cells to its role in human cells though indeed a recent study of HIV-1 integration in wild-type and mutant mouse cells suggest that it is a significant player in virus integration [27]. It is interesting to note that when we aligned the protein sequences of the mouse and human LEDGF proteins, we observed that the proteins share 92% identity overall and the integrase binding domain of hLEDGF identified by Cherepanov [32] shares 100% consensus with the corresponding region in mLEDGF (data not shown).

### Chromatin binding and transcriptional activators

One category of proteins isolated in the screens is of particular interest because it includes DNA binding and chromatin modification factors. Enhancer of Zeste homolog 1, (Enx-1/Ezh2), is a member of Polycomb repressive complex 2 (PRC2). The isolation of a member of this class of proteins is not without precedence: one of its PRC2 partner proteins, embryonic ectodermal development factor (EED), has been identified as an interactor with other retroviral proteins. EED was isolated in a yeast two-hybrid screen with HIV-1 MA as bait and later shown to interact with HIV-1 IN [33,34]. The interaction with HIV-1 IN led to an increase in integration in vitro [34]. Another yeast two-hybrid screen using HIV-1 Nef as bait recovered EED from a Jurkat cDNA library [35]. Analyses of the interaction between Nef and EED revealed that Nef mimics an integrin receptor signal and translocates EED from the nucleus and relocalizes it to the plasma membrane, resulting in an increase in Tat mediated HIV transcription [35]. Enhancer of zeste [E(Z)] and extra sex combs (Esc), the drosophila homologs of mammalian Enx-1 and EED respectively, are part of the same repressive complex in both drosophila and mammalian cells. In fact, Enx-1 and EED interact both in vitro in yeast and in vivo in mouse cells [36]. Intriguing questions are whether or not Enx-1 is also translocated to the plasma membrane in a complex with EED, and whether both proteins play similar roles in the viral life cycle or have a comparable effect independently on viral infectivity and integration. Although none of the studies cited above investigated an interaction between EED and MoMLV IN, the isolation of Enx-1 in our screen, and our finding that it also interacts with HIV IN suggests the intriguing possibility of a role for the PRC2 chromatin repressor complex in the viral life cycle.

Acute lymphocytic leukemia gene 1 fused from chromosome 9 (*AF9*), also known as mixed lineage leukemia translocated to chromosome 3 homolog (*Mllt3*) is frequently found in balanced translocations with the mixed lineage leukemia gene (*MLL*), a trithorax homolog, in acute myeloid leukemia cells. In mice, MLL is required for normal embryogenesis and likely regulates Hox gene expression by binding to promoter sequences [37]. The precise function of AF9 is unknown, but it has been proposed as a transcriptional activator as it contains a serine- and proline-rich domain, as well as a nuclear localization signal, consistent with such a role. Null *af9 *mice exhibit homeotic transformations and perinatal lethality, suggesting that AF9 may be a master regulator of *Hox *genes [38]. The C-terminus of AF9 interacts with the mouse and human homologs of the Drosophila Polycomb group protein Pc3, and with the BCL6 corepressor BcoR: both Pc3 and BcoR normally act to repress transcription [39,40]. In this report, we isolated four clones of AF9 in our screens and we show that at least one of these clones interacts with HIV IN and MoMLV IN in yeast and in the in vitro binding assays. An intriguing question raised is whether disruption of the opposing activities of Polycomb and Trithorax proteins will reveal a role for these proteins in retroviral integration, given that Trithorax proteins are transcriptional activators and Polycomb proteins are transcriptional repressors.

In our screens, the largest number of clones isolated corresponded to the cDNA for bromodomain containing protein 2 (Brd2/fsrg1/RING3) (nine isolates). Proteins that contain bromodomain motifs function in the regulation of chromatin and in epigenetics [41]. The bromodomain is found in the majority of histone acetyltransferases and in transcriptional activators, and derives its name from the *Drosophila *brahma protein in which the motif was initially identified [42]. Brd2 functions as a transcriptional co-activator and as a nuclear-localized kinase [43]. Recent studies have identified a Brd2 complex that contains, among others, E2F (E2 promoter binding factor), histones, HDAC11, CBP, p300, Cyclin A2, TAF_II_250, and Swi/Snf chromatin remodeling complex member Brg-1 [41,44]. In the Denis et al. studies, overproduction of Brd2 led to elevated Cyclin A transcription and a presumed destabilization of the cell cycle, as Brd2 was associated with the *cyclin A *promoter at both the G_1 _and S phases [41]. In addition, Brd2 was shown to interact with the chromatin-binding domain in the Kaposi's sarcoma-associated Herpes virus (KSHV) latency-associated nuclear antigen 1 (LANA-1) to modulate transcription and episomal DNA replication [45]. LANA-1 may interact with Brd2 to tether the KSHV genome to mitotic chromosomes in a manner similar to that observed between the Bovine papillomavirus (BPV) E2 protein and Brd4 [46]. Although the observed interaction between Brd2 and HIV-1 IN in yeast was weaker than its interaction with MLV IN, the finding that the Brd2-HIV IN in vitro interaction is apparently equal in intensity to that observed for MLV IN suggests that this protein may play a role in the integration of both retroviruses. Baz2b is another bromodomain family member identified in our screen, whose precise function remains to be elucidated [47]. Baz2b exhibits the same behavior as that observed for Brd2 in our assays: it displays a weaker interaction in yeast with HIV IN than that observed for MLV IN, but an in vitro binding apparently equivalent to that observed for MLV IN.

B-ATF is a member of the AP-1/ATF superfamily of transcription factors [48] and its expression in human and mouse is tissue specific, primarily limited to hematopoetic tissues and cells [49]. B-ATF contains a basic Leucine zipper motif, does not homodimerize, does not contain a functional transcription activation domain, and does not dimerize with Fos, but does form heterodimers with the Jun family proteins (c-Jun, JunD and JunB) to bind Activator protein-1 (AP-1) consensus DNA sites [49]. B-ATF is a natural dominant-negative regulator of AP-1 mediated transcription, acting as a non-activating competitor for c-Fos in the AP-1 dimer to reduce cell growth [49]. Ectopically expressed B-ATF reduced transformation by *v-fos *and *H-ras *oncogenes in mouse cells [49]. Rasmussen et al. [50] identified T-cell lymphoma-specific MoMLV integrations at the *Fos/Jdp2/Batf *locus in mouse cells. The B-ATF clone isolated in our screen did not interact with HIV-IN in yeast, but a role for this factor in transformation by MoMLV should be investigated.

Zinc finger p38 is a transcriptional activator that contains seven Cys_2_His_2 _type zinc fingers, a SCAN box (SRE-ZBP, CTfin51, AW-1 (znf174), and Number 18), also known as the Leucine rich region, and a novel N-terminal domain [51]. The SCAN domain may be a protein-protein interaction motif, as mammalian two-hybrid studies have identified this region as capable of transcriptional activation [52,53]. The finding that our Znfp38 clone interacted with both MLV IN and HIV-1 IN both in yeast and in vitro, suggests a role for this transcription factor in the life cycle of both retroviruses.

### DNA repair proteins

A surprising find was the isolation of Ku70/XRCC6, the 70 kD subunit of the Ku70/Ku80 thyroid autoantigen, also known as the Ku heterodimer. Ku70 was initially identified by the isolation of an abundant antibody found in patients with autoimmune thyroid disease and lupus erythematosus [54]. The Ku86 heterodimer has ATP-dependent DNA helicase activity [55], is thought to be the first protein to bind to a DNA double strand break [56], functions as a sliding clamp on DNA and recruits DNA-PK_cs_, DNA polymerases, and ligases to the site of damage [57] in a manner similar to the mechanism employed by PCNA [58]. The Ku heterodimer participates in the non-homologous DNA end joining (NHEJ) pathway of DNA repair [59], in V(D)J recombination, and with Telomere repeat factor 2 (TRF2) to suppress homologous recombination of telomeres between sister chromatids [60]. Additional studies have identified a role for the NHEJ complex in Ty1 retrotransposition [61] and in retroviral integration [62,63]. The isolation of Ku70 in our screen and the in vitro binding data suggest that this protein may play a direct role in integration for both MLV and HIV-1.

Flap endonuclease-1 (Fen1), or RAD two homolog-1 (Rad27 or RTH1) is a structure-specific 5' endo/exonuclease that functions in the maintenance of genome stability, long-patch base excision repair, NHEJ, and the resolution of Okazaki fragments in lagging strand DNA synthesis [64]. Deletions of Fen-1/Rad27 in yeast cells lead to a high frequency of chromosome loss and an increased rate of recombination [64]. The C-terminus of Fen-1 interacts with the transcription coactivator p300, which acetylates Fen-1 [65], and has been implicated in retroviral integration [66,67]. Although Fen-1 was identified in a yeast two-hybrid screen as an interaction partner of Friend virus susceptibility 1 protein (Fv-1) (Subarna Bhattacharyya, unpublished data), the report of Rumbaugh et al. [68] demonstrating the involvement of Fen-1 in the processing of HIV-1 integration intermediates [68] prompted us to examine a possible direct interaction between Fen-1 and the integrases of MoMLV and HIV-1. The in vivo and in vitro interactions observed in our report support a direct interaction between Fen-1 and the two integrases, suggesting that experiments designed to delineate the precise role of Fen-1 in the DNA repair step of integration in vivo should be pursued.

### RNA binding proteins

Spliceosomal small ribonucleoproteins (snRNPs) are major components of the mRNA splicing machinery and each snRNP is comprised of one or two small nuclear RNAs (snRNAs) bound to a set of RNA-binding proteins, called Sm proteins (SmB/B', SmD1, SmD2, SmD3, SmDE, SmF, and SmG) [69]. The Sm proteins bind to a highly conserved uridine rich sequence on each snRNA called the Sm site. Sm cores are assembled in vivo onto snRNAs by the SMN complex [69]. *Survival motor neuron *(*SMN*) is the gene for spinal muscular atrophy (SMA) whose disruption in mouse embryos leads to massive cell death and early embryonic lethality [70]. SMN is part of a large complex with at least six to seven Gemin proteins (Gemins 2–8) that function to organize snRNPs [69]. SMN interacts directly with Gemins 2, 3, and 8 [71]. Reduction of SMN levels by an SMA-causing mutation leads a decrease in the relative amounts of Gemins as part of the SMN complex [71]. A recent report identified Gemin2 as an HIV-1 integrase interactor by yeast two-hybrid screening [72]. The Hamamoto [72] report used siRNA to downregulate Gemin2 and SMN in cells subsequently infected by HIV-1, showing that disruption of these proteins blocked HIV-1 infection, and Gemin2 disruption reduced viral DNA copy number, 2-LTR circle accumulation, and proviral integration [72]. Interestingly, SMN also interacts with snRNPs U1, U2 and U5. The U2snRNP associated factor U2AF^26 ^and U5snRNP were also isolated in our screen, suggesting the possibility of an interaction between the incoming viral RNA and the spliceosomal network, or that integrase may co-opt these factors for downstream viral functions.

The U2 snRNP is an essential component of the spliceosome and binds to the pre-mRNA branch site by base-pairing with the complementary RNA sequence of the U2 snRNA [73]. U2 snRNP interacts with the U1 snRNP which binds to the 5' splice site, and a complex of U1 snRNP/U2 snRNP/pre-mRNA recruits the U4/U6/U5 snRNPs to form an active spliceosome [73]. The core 12S U2 snRNP binds splicing factor 3b (SF3b), to form a pre-mature 15S U2 snRNP [74]. In turn, this complex binds SF3a to form a mature 17S snRNP, which interacts with nucleotides upstream of the branch site within the intron [74]. Splicing factor 3a subunit 3 (also known as SF3a3, Sf3a60 and Spf3a3) is the mammalian homolog of *S. cerevisiae *PRP9 and is a C2H2- type zinc finger protein required for the core complex assembly [75]. The SF3a complex is composed of SF3a60, SF3a66 and SF3a120, of which we have isolated the 60 kD subunit (SF3a3) in our screen. In addition, we isolated the SF3b2 subunit of SF3b in our screen, which interacts directly with SF3a. We also isolated the factors U2AF^26^, U5 snRNP, and SMN as described above. Would the incoming virus interact with these proteins? The isolation of these core spliceosome components suggests that a new perspective on integrase-host factor interactions may be required upon further analysis of these factors.

### Other factors

Peroxisome proliferative-activated receptor gamma coactivator-1α, PGC-1α (formerly PGC-1), is a nuclear hormone receptor that coordinates diverse organ- and cell-specific transcription programs in response to stress stimuli [76]. Two additional genes in the family have been identified, PGC-1-related coactivator (PRC) and PGC-1β (PERC/ERRL-1) [77,78]. Each of the genes share domain organization: an N-terminal region containing a nuclear hormone receptor interacting motif, an LXXLL coactivator motif, an RS-rich domain, and a C-terminal RNA binding motif [77,78]. Both PGC-1α and PRC interact via their C-terminal domains with nuclear respiratory factor 1 (NRF-1), a transcription factor that activates a number of mitochondrion-related genes. In addition, NRF-1 has been implicated in biosynthetic pathways of two rate-limiting enzymes in purine nucleotide biosynthesis by the presence of functional NRF-1 binding sites in their promoters: the CXCR4 chemokine receptor, and the human poliovirus receptor CD155 [79-81]. PRC enhances NRF-1-dependent transcription in vitro and in vivo [77]. Unlike PGC-1α, PRC is ubiquitously expressed in all tissues, but is cell cycle regulated as cells arrested in G_0 _exhibit barely detectable levels of mRNA or protein, but expression levels return to detectable levels after addition of serum [77]. The PRC clones in our study all contain the C-terminal RNA recognition motif, and the clone examined in our assays interacted with MLV IN in vivo and in vitro and exhibited a moderate interaction with HIV IN in these studies.

Our screen identified Radixin, a member of the ERM (Ezrin-Radixin-Moesin) family of proteins, as an interactor with MoMLV IN and HIV-1 IN. This protein family regulates cortical structure and has a role in Rho and Rac signaling pathways [82]. The ERM proteins exhibit approximately 75% amino acid sequence identity between them and each protein contains a domain known as the band 4.1 ERM domain (the four-point one ezrin radixin moesin, or FERM domain), which comprises about 300 residues of the amino-terminal region in each protein, and binds the plasma membrane. Each ERM protein also contains a stretch of approximately 30 residues in their carboxyl-terminal domains that bind to F-actin. Expression of these proteins is often cell type- and organ- specific: it is of interest to note that although some T-cell lines do not express detectable levels of radixin, the cDNA corresponding to radixin was isolated from a T-cell library in our screen. Radixin is activated by the unmasking of FERM domains by the binding of phosphatidylinositol 4, 5 bisphosphate (PIP_2_) [83]. Growth factor-induced phosphorylation at C-terminal threonines by Rho-associated kinase, protein kinase C (PKC)-α, or PKC-θ stabilizes the unmasked ERM proteins in an open form, thus regulating binding to actin [84]. Thus far, none of the ERM proteins has been identified as a *bona fide *tumor suppressor except Merlin (moesin-ezrin-radixin-like protein), which was identified as the gene for neurofibromatiosis-2 (NF-2) [83]. Recently, overexpression of Moesin was found to inhibit infection of both HIV and MLV viruses at a step prior to the initiation of reverse transcription [85]. In addition, endogenous levels of Moesin inhibited viral replication [85]. Investigation of a possible role for Radixin in the integration reaction may yield new insights into a regulatory function for another member the ERM family of proteins in retroviral infectivity.

## Conclusion

There are many steps during retroviral infection that may afford opportunities for the viral integrase to interact with host factors: following cytoplasmic entry, during reverse transcription, at or during nuclear entry, prior to and after genomic integration, during transcription of viral RNA, or even during virus gene expression and virion production. As different retroviruses appear to favor different integration target sites, a preference for specific host factors as chromatin tethers or for targeting the viral genome to specific sites may be influenced by target site preferences specific to the virus [86,87].

In summary, we used MoMLV integrase as bait in a series of yeast two-hybrid screens to isolate 27 putative integrase interacting proteins. These proteins also interacted to varying degrees with HIV-1 IN in two-hybrid assays. Seventeen of these proteins were examined in MBP-GST binding assays with MBP fusions of MLV and HIV integrases and the clones interacted to varying degrees with MLV IN and HIV IN in these assays. The isolation of chromatin remodeling factors (Enx-1, AF9, Brd2, Baz2b), DNA repair proteins (Ku70 and Fen1), transcriptional activators (B-ATF3, TFIIE-β, PRC, Ankrd49, Znfp15 and Znfp38) and several distinct components of the spliceosome (U5 snRNP, U2AF^26^, SMN, SF3a3, SF3b2, SLU7) suggest new pathways to explore in the analysis of integrase host factor interactions. Many of the proteins identified in the screen are logical interaction partners for integrase, and the validity of the interactions are supported by other studies (Ku70, Fen-1 and SMN). In addition, the finding that Brd2 interacts with KHSV protein LANA-1 raises the intriguing possibility that there may be common host proteins used by viruses other than retroviruses. We originally undertook this screen to obtain potential host factors that might affect integration target site selection. The yeast two-hybrid screens described herein have generated a wealth of putative interacting proteins that merit further investigation. We make no strong assumptions that each of the proteins presented in this work will exhibit profound effects on the integration reaction in vitro, nor in vivo. We present a group of potential interaction partners for Moloney and HIV-1 integrases that we hope will provide new avenues to explore in our efforts to understand interactions between viral integrases and host proteins.

## Methods

### Yeast strains

The *Saccharomyces cerevisiae *strain CTY10-5d (*MATa ade2 trp1-901 leu2-3,112 his3-200 gal4 gal80 URA3::lexAop-lacZ ura3-52*), a generous gift from Dr. Rolf Sternglanz, State University of New York at Stonybrook, was the strain used to screen the cDNA libraries and to examine the interactions between MoMLV IN deletions and the putative interacting proteins identified in the screens. We also used CTY10-5d to examine interactions between HIV-1 IN and a subset of clones identified in the screen. SFY526 (*MATa ura3-52 his3-200 ade2-101 lys2-801 trp1-901 leu2-3,112 can*^*r*^*gal4-542 gal80-538 URA::GAL1*_*UAS*_*-GAL1*_*TATA*_*-lacZ*), a generous gift from Dr. Michael Stallcup (University of Southern California, Los Angeles, CA), was used to examine weaker interactions of clones obtained in the screen.

### Yeast two-hybrid bait shuttle vectors

Moloney murine leukemia virus integrase was subcloned from the plasmid pNCA, which contains the entire proviral genome of MoMLV. The PCR fragments corresponding to the MoMLV integrase inserts were subcloned into the EcoRI and SalI sites of the plasmid pSH2-1 [88], using the primer pairs listed in Table S2 in additional file 3, resulting in the plasmid herein known as pSH2-mIN. This plasmid contains a truncated lexA DNA binding domain and allows fusions to the carboxyl-terminus of lexA. We also constructed a version of this plasmid containing a six glycine linker at the N-terminus of IN, pSH2-mIN 6G (see Table S2 in Additional file 3 for oligos used). The full-length lexA reporter (amino acids 1–202, a derivative of pEG202) plasmid pNlexA (constructed by M. Sainz and S. Nottwehr and a gift from Erica Golemis, Fox Chase Cancer Center, Philadelphia, PA) was used to generate an amino terminal lexA fusion of MoMLV integrase. The mIN insert was subcloned into the EcoRI and BamHI sites by PCR (Expand High Fidelity, Roche) using the primer pairs listed in Table S2 in Additional file 3, generating plasmid mIN-pNlexA. MoMLV Integrase was subcloned into the GAL4 DNA binding domain vector pGBKT7 (Clontech, USA) by insertion of the EcoRI-SalI integrase fragment from pSH2-mIN to generate pGBKT7-mIN. The pSH2-HIV-1 integrase construct (herein called pSH2-hIN) was described previously [21], and the integrase insert was subcloned into pGBKT7 using the BamHI-SalI insert from pSH2-hIN to generate pGBKT7-hIN. The cDNA corresponding to *Mus musculus *LEDGF was subcloned by PCR from MGC:57990, IMAGE:6400529, Genbank accession number BC043079/BU702373 in pYX-ASC (Invitrogen Clones, USA) into pSH2-1, pGBKT7 and pGADNOT [20] using the primers listed on Table S2 in Additional file 3. The insert from pMA424-MoMLV Gag [89] was subcloned into the following vectors for use as controls: pGBKT7, pGADNOT, and pACT2. All yeast plasmids, including library plasmids, were sequenced using the following oligonucleotides:5'ADH: 5'-GTTTGCCGCTTTGCTATCAAG-3' and 3'ADH: 5'-GTTTTAAAACCTAAGAGTCAC-3'. All constructs were also sequenced with internal oligonucleotides.

### Yeast protein isolation

Single colonies corresponding to each of the bait and control plasmids were isolated and grown in 5 ml minimal media lacking either His or Trp at 30°C until the O.D._600 _reached 0.7. For processing, the pellets were thawed on ice and resuspended in 200 μl Yeast Extraction Buffer (25 mM Tris-HCl pH 7.5, 150 mM NaCl, 1 mM EDTA, 1 mM PMSF, 7 mM β-mercaptoethanol (β-Me), 10% glycerol). The cell suspensions were lysed using glass beads (425–600 micron, Sigma) by vortexing 30 seconds, followed by a 30 second incubation on ice; this procedure was repeated 5 times, after which the tubes were centrifuged for 15 minutes at 14,000 rpm, 4°C. The supernatant was transferred to chilled tubes and the beads were washed with 100 μl of fresh extraction buffer, followed again by centrifugation. The resulting supernatant was pooled with the first and diluted 1:1 with 2X Protein Sample buffer (0.125 M Tri-HCl pH 6.8, 2% SDS, 20% glycerol, 0.1 mg/ml Bromophenol blue, 5% β-Me) and loaded to 10 or 12% SDS-PAGE gels for Western blotting, transferred to PVDF membranes (Immobilon-P, IPVH00010, Millipore), and probed with anti-lexA (R990-25, Invitrogen), or anti-GAL4 DB (RK5C1, Santa Cruz Biotechnology).

### Library plasmids and screens

The WEHI-3B cDNA library, in the GAL4 activation domain vector pGADNOT, was described previously [30]. The T-cell mouse cDNA library in λ ACT2 was a generous gift from Dr. Stephen J. Elledge, Harvard University. The *Escherichia coli *strain LE392 (*F*^-^*e14*^- ^(McrA^-^) *hsdR514 *(r_K_^- ^m_K_^+^) *supE44 supF58 lacY1 or •(lacIZY)6 galK2 galT22 metB1 trpR55*), a gift from Dr. Max Gottesman, Columbia University, was used to titer the λACT2 phage library and the strain BNN132 (JM107/λKC, kan^*r*^, lambda lysogen containing the *cre *gene (*F' traD36 lacI*^*q *^*• (lacZ)M15 proA*^-^*B*^+^/e14^-^(McrA^-^) *•(lac-proAB) thi gyrA96 (Nal*^*r*^*) endA1 hsdR17*(r_k_^-^m_k_^+^)*relA1 supE44*), also a gift from Dr. Elledge, was used to convert the λ ACT2 library to the plasmid library in pACT2, using the method described by Durfee et al. [90]. Clonal expansions of all bait and control plasmids were performed in the *E. coli *strain DH5α prepared by standard CaCl_2 _transformation procedures.

Three independent yeast two-hybrid screens were performed using two cDNA libraries, the pGADNOT-WEHI-3B cDNA library described above, and the pACT2-T-cell cDNA library. For all screens, a single CTY5-10d colony bearing a pre-transformed lexA-integrase fusion plasmid was transformed with 30 μg library DNA into 500 ml log phase cultures by the Lithium Acetate method of Schiestl and Gietz [91]. Transformants were plated on 15-cm synthetic complete media plates lacking Histidine and Leucine and allowed to grow for three days, after which time the colonies were transferred to nitrocellulose membranes (Schleicher and Schuell), stored at -80°C for 2 hours to overnight. The nitrocellulose membranes were thawed at room temperature and X-gal colony lift assays were performed at 30°C and monitored every hour for six hours to overnight for the development of blue colonies indicative of β-galactosidase activity [92]. Blue colonies were isolated and streaked to fresh SC-His-Leu plates and lifted onto nitrocellulose membranes and assayed again in the X-gal colony lift assay. One-half of three blue colonies from each plate were patched to master plates for preparation of stocks, and the other half was transferred to 5 ml of SC-Leu media and incubated at 30°C overnight for plasmid rescue. Yeast DNA was extracted using the Zymoprep Yeast Plasmid Minipreparation I Kit (Zymo Research, Orange, CA) with the following modification: the DNA pellets were washed three times in 70% ethanol. A combined total of 1.2 × 10^6 ^transformants were analyzed in the three screens.

Rescued yeast DNAs were transformed into *E. coli *strain KC8 by electroporation using standard procedures. The transformants were plated on M9-Leu ampicillin selective plates and a minimum of six colonies from each putative clone were isolated and amplified. The rescued plasmids were then retransformed into CTY10-5d, bearing either the pSH2-mIN or mIN-pNlexA bait plasmid, and the X-gal colony lift assay repeated. Plasmids DNAs corresponding to positive clones, as indicated by blue color in the lift assay, were sequenced. The positive clones identified in the screen were also transformed into CTY10-5d bearing pSH2-hIN and tested in the colony lift assay. The rescued, sequenced, positive clones were also transformed into SFY526 strains bearing the empty vector pGBKT7, pGBKT7-mIN or pGBKT7-hIN plasmids and tested in the colony lift assay.

### MoMLV IN deletion constructs

Domain or motif deletions of MoMLV integrase were constructed by PCR (Expand High Fidelity, Roche) using the proviral plasmid pNCA as template. The deletions were engineered with 5' EcoRI and 3' SalI sites for subcloning into pSH2-1. The constructs retained the following regions: Zinc binding motif only (pSH2-mIN-Zn); Zinc binding motif and catalytic domain (pSH2-mIN-ZnDDE); catalytic domain (pSH2-mIN-DDE); catalytic domain and C-terminal domain (pSH2-mIN-DDECOOH); and the C-terminal domain only (pSH2-mIN-COOH).

### Protein expression vectors

The pmalc2-MoMLV integrase plasmid used for protein expression studies was constructed by subcloning the EcoRI/SalI insert from pSH2-MLV IN into the maltose fusion vector pmalc2 (New England Biolabs) to generate pmalc2-mIN and the HIV-1 IN plasmid was constructed by subcloning a BamHI-XhoI insert generated by PCR from pSH2-HIV-1 IN, and ligating it into the BamHI-SalI site of pmalc2, to generate pmalc2-hIN. The pmalc2-MoMLV IN and pmalc2-HIV-1 IN constructs were transformed into *E. coli *strain TB1 or DH5α for expression. The library inserts were subcloned into the vector pGEX2TPL, a laboratory modified version of the glutathione-S transferase fusion vector pGEX2T (Pharmacia/GE healthcare), into which an extensive polylinker was inserted, using the following sites for the various WEHI-3B/library inserts: for AF9, TFIIE-β, Brd2, B-ATF, and PRC: XbaI/BglII; for Zinc finger p38, Ankyrin repeat domain 49, KIF3A, Baz2b, and U5 snRNP: SpeI/BglII; and for Enx-1, and Fen-1: AvaI/BglII. The pACT2 T-cell library inserts for U2AF^26^, Tata binding protein Activator of Basal Transcription-1, Brd2, Ran binding protein 10 were subcloned using the XhoI site. The inserts for Ku70, PRC, and SF3a3 were subcloned by PCR using oligonucleotides designed with BamHI/EcoRI sites; or for Radixin and TFIIE-β using BamHI/XhoI sites (Table S2 in Additional file 3). The resulting GST fusion plasmids containing the yeast two-hybrid inserts were transformed into BL21 for expression. Protein expression for all bacterial strains was induced when the optical density at 600 nm reached 0.8 by the addition of 100–200 μM or 400 μM isopropyl-β-D-thiogalactoside (IPTG) for pGEX2T-PL or pmalc2 constructs respectively, in 50 ml or 100 ml cultures for 3–5 hours at 37°C, or at 28°C for pGEX2TPL-Ku70, -PRC and -Radixin. All induced cultures were collected by centrifugation at 4,000 rpm for 15 minutes, washed twice in Buffer A (50 mM Tris-HCl, pH 8, 200 mM NaCl, 5 mM EDTA, 10 mM 2-mercaptoethanol, 1 mM phenylmethylsulfonyl fluoride (PMSF), protease inhibitors (Roche), or Buffer C (100 mM Tris-HCl pH 8, 200 mM NaCl, 5 mM EDTA, 10 mM dithioretol (DTT), protease inhibitors (Roche), 1 mM PMSF) and the pellets stored at -80°C until processing.

### MBP – GST in vitro binding assays

Pellets for pmalc2-MoMLV IN, pmalc2-HIV IN, or the pGEX2T-PL two-hybrid fusion expression plasmids were thawed on ice, resuspended in Buffer A or C plus 0.5 mg/ml lysozyme and incubated one hour at 4°C on a rocking platform. Pellets were sonicated and the crude lysates were centrifuged 30 min. at 13,000 rpm, 4°C, the clarified supernatants collected, glycerol added to 20%, aliquoted in 100 μl volumes, and flash frozen or used immediately. Expression of GST fusion proteins was examined following manufacturer's instructions (GE Healthcare). For the amylose resin binding assay, 2–25 μl of each maltose fusion protein lysate, depending on expression levels, in a total volume of 200 μl was mixed with 200 μl of pre-equilibrated amylose resin (prepared according to manufacturers instructions, New England Biolabs) that was pre-equilibrated in Buffer A or Buffer C. The binding reactions were incubated at 4°C for one hour and washed four times in Buffer A or Buffer C. For each MBP fusion binding of library-GST fusion protein lysate, 50 – 100 μl of each GST fusion lysate was added to the washed MBP fusion protein binding reaction and incubation was continued for one hour at 4°C on a rocking platform. The MBP-GST complexes were then washed four times in Buffer A or Buffer C containing 0.1–0.3% IGEPAL CA-630 (I-3021, Sigma), and a total of four elutions were performed as follows. The washed complexes were incubated overnight at 4°C in 200 μl, 15 mM maltose prepared in Buffer C without detergent, followed by centrifugation for 10 minutes at 4,000 rpm. This elution step was repeated three times with 50 μl of 15 mM maltose in Buffer C (instead of 200 μl) and incubated at room temperature for one hour each time for a total of four elutions. The eluates were pooled and 20 – 25 μl was electrophoresed on 10 or 12% SDS-PAGE gels (Nu-Sep, N.A. Austell GA) and transferred to PVDF membranes (Immobilon-P, IPVH00010, Millipore) for Western blotting by standard procedures. The membranes were incubated successively with GST antibody at 1:2000 dilution (MMS-112P, Covance) and MBP antibody at 1:5000 dilution (93–5100, Zymed), stripping between each probe. Secondary antibodies were horseradish peroxidase (HRP)-conjugated anti-mouse (NA931, GE Healthcare) used at 1:10,000 dilution in 6% non-fat dry milk/TBST and were visualized with chemiluminescent substrate (Perkin-Elmer Western Lightning).

### Nuclease treatment of MBP and GST lysates

Each *E. coli *lysate from strains expressing MBP- or GST-fusions were treated independently with 2 μl (4U) of Turbo DNA-*free *(Applied Biosystems) and 2 μg of RNase (Roche) in Turbo DNA-*free *reaction buffer in a total volume of 50–100 μl per reaction and incubated at 25°C for 30–60 minutes. Samples of treated and untreated lysates were removed and electrophoresed in 1.5% agarose gels and stained with ethidium bromide to determine the presence or absence of nucleic acids (see Figure S1 in Additional file 2). Following nuclease treatment, the MBP-integrase and GST-fusion lysates were mixed, and binding assays performed as previously described. The nuclease treated binding reactions were electrophoresed on 10% SDS-PAGE gels (NuSep) and transferred for Western blotting and probed successively with anti-GST and anti-MBP antibodies in the same manner as described in this report.

## Competing interests

The authors declare that they have no competing interests.

## Authors' contributions

BS designed and performed all of the experiments, drafted and edited the manuscript, SPG reviewed and edited the manuscript. Both authors approved the manuscript.

## Supplementary Material

Additional file 1 Table S1. **Results of the T-cell library yeast two-hybrid screen**. number of colonies screened in T-cell library. Click here for file

Additional file 2 Figure S1. **Nuclease treatment of MBP and GST lysates**. Ethidium bromide-stained agarose gel of nuclease treated lysates. Click here for file

Additional file 3 Table S2. **Oligonucleotides used in this study**. Oligonucleotides used to construct the bait and protein expression constructs. Click here for file
