# Supplementary material for: Host proteins interacting with the Moloney murine leukemia virus integrase: Multiple transcriptional regulators and chromatin binding factors
Source: Retrovirology. 2008 Jun 13;5:48. doi: 10.1186/1742-4690-5-48 (PMC2481268; doi:10.1186/1742-4690-5-48)
Supplement: Additional file 1 — Table S1. Results of the T-cell library yeast two-hybrid screen. number of colonies screened in T-cell library. [file 1742-4690-5-48-S1.pdf]

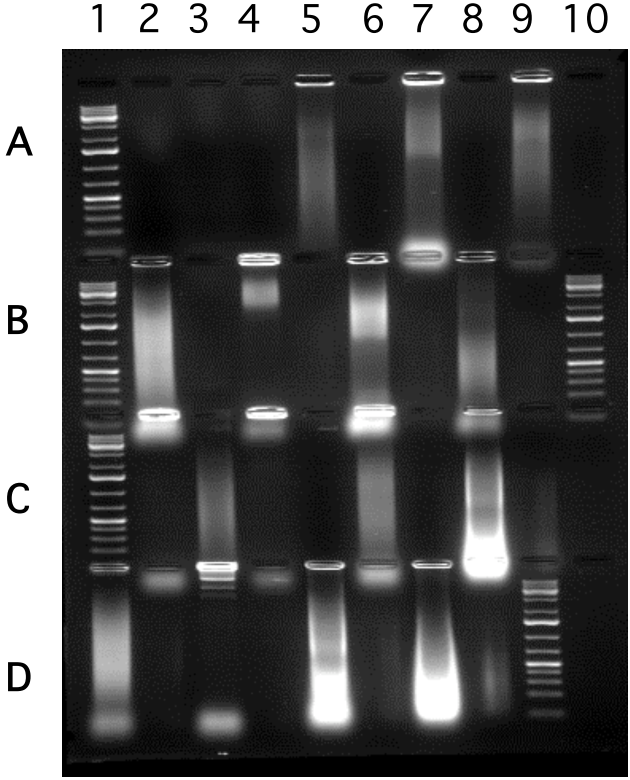


**Figure S1.** Nuclease treatment of MBP and GST lysates. The untreated and treated lysates were electrophoresed on 1.5% agarose gels. The treated lysates were mixed and used in the pull-down binding assays. **(A)** Samples of nuclease treated MBP and GST lysates prior to and following binding reactions. Lane 1, DNA ladder; treated eluates following pull-down assays: lane 2, MBP + GST; lane 3, mIN + GST; lane 4, hIN + GST. Lysates before binding reactions: lanes 5 and 6, untreated and treated MBP; lanes 7 and 8, untreated and treated mIN; lanes 9 and 10, untreated and treated hIN. **(B)** Lysates prior to binding reactions, treated and untreated. Lane 1, DNA ladder; lanes 2 and 3, untreated and treated GST; lanes 4 and 5, untreated and treated Brd2; lanes 6 and 7, untreated and treated AF9; lanes 8 and 9, untreated and treated Ankr49; lane 10, DNA ladder. **(C)** GST lysates prior to binding reactions. Lane 1, DNA ladder; lanes 2 and 3 untreated and treated PRC; lanes 4 and 5, untreated and treated Baz2b; lanes 6 and 7 untreated and treated ABT1; lanes 8 and 9 untreated and treated Radixin. **(D)** Lanes 1 and 2, untreated and treated Fen-1; lanes 3 and 4, untreated and treated Enx-1; lanes 5 and 6, untreated and treated TFIIE-β; lanes 7 and 8, untreated and treated Ku70; lane 9, DNA ladder.
