# Supplementary material for: Host proteins interacting with the Moloney murine leukemia virus integrase: Multiple transcriptional regulators and chromatin binding factors
Source: Retrovirology. 2008 Jun 13;5:48. doi: 10.1186/1742-4690-5-48 (PMC2481268; doi:10.1186/1742-4690-5-48)
Supplement: Additional file 2 — Figure S1. Nuclease treatment of MBP and GST lysates. Ethidium bromide-stained agarose gel of nuclease treated lysates. [file 1742-4690-5-48-S2.pdf]

Table S1. Results of the T-cell library yeast two-hybrid screen

	T-cell library			
	Pool 1	Pool 2	Pool 3	Pool 4
**Bait plasmid**	Colonies screened	Colonies screened	Colonies screened	Colonies screened
pSH2-MoMLV IN	455,298	215,291	286,469	-
MoMLV IN-pNlexA	-	52,749	43,169	50,170
Total screened/pool	455,298	268,040	329,638	50,170
Number of clones isolated	19	2	3	1
No. clones isolated, T-cell screens: 25				
No. colonies screened: 1,103,146				

The DNA binding domain lexA bait vectors pSH2-MoMLV IN and MoMLV IN-pNlexA were used to screen the murine T-cell library in pACT in the lexA reporter strain CTY10-5d. 1.1×10^6 colonies were screened, yielding 25 interacting proteins from the indicated independent pools of transformants in this library.
