# Supplementary material for: Host proteins interacting with the Moloney murine leukemia virus integrase: Multiple transcriptional regulators and chromatin binding factors
Source: Retrovirology. 2008 Jun 13;5:48. doi: 10.1186/1742-4690-5-48 (PMC2481268; doi:10.1186/1742-4690-5-48)
Supplement: Additional file 3 — Table S2. Oligonucleotides used in this study. Oligonucleotides used to construct the bait and protein expression constructs. [file 1742-4690-5-48-S3.pdf]

Table S2. Oligonucleotides used in this study

Insert	Destination plasmid	Primer
MoMLV integrase	pSH2-1	BSO1, EcoRI: 5'GGAATTCATAGAAAATTCATCACCCTACACCTCAGAA 3'
MoMLV integrase	pSH2-1	BSO7, 3'SalI: 5'TGCGGTCGACTTAGGGGGCCTCGCGGGTTAACC3'
MoMLV integrase	pSH2-1	BSO2, EcoRI 6gly: 5' GGAATTCGGGGGCGGAGGCGGGGGCATAGAAAATTCATCACCC 3'
MoMLV integrase	pNlexA	BSO26, EcoRI: 5' CGGAATTCGGCCGCCATGATAGAAAATTCATCACCCTACACC 3'
MoMLV integrase	pNlexA	BSO29, 3' BamHI: 5' GAACGGGATCCCGGGGGCCTCGCGGGTCAGCCT 3'
HIV-1 integrase	pmalc2	BSO37, BamHI: 5' GCGGATCATGTTTTAGATGGAATAG 3'
HIV-1 integrase	pmalc2	BSO39, 3'XhoI: 5' CCGCTCGAGCTAACCCAATTCTGAAAATGGATAAACAGC 3'
mmLEDGF	pSH2-1	BS057, EcoRI: 5' CGGAATTCATGACTCGCGATTTCAAACCTGGA 3'
mmLEDGF	pSH2-1	BSO58, 3' SalI: 5' CTGAAAGAGTCTACACTAGATAACTAGGTCGACCAATTC 3'
mmLEDGF	pGADNOT	BSO 60, BamHI: 5' GCGGATCCGCATGACTCGCGATTTCAAACCT 3'
mmLEDGF	pGEX2T-PL	BSO 61, 5' BamHI: 5' CGTGGATCCATGACTCGCGATTTCAAACCTGGA 3'
mmLEDGF	pGEX2T-PL	BSO62, 3' XhoI: 5' CCGCTCGAGCCTAGTTATCTAGTGTAGACTCTTTCAG 3'
Ku70	pGEX2T-PL	5'BamHI: 5' CGCGGATCCCCAAGTGAGCAAACC3'
Ku70	pGEX2T-PL	3'EcoRI: 5' CGGAATTCCAGTTCACTGGGAGA 3'
PRC	pGEX2T-PL	5' BamHI: 5'CGCGGATCCGAGTTCACCTTGGCT3'
PRC	pGEX2T-PL	3' EcoRI: 5'CGGAATTCGCTCGAGAGTTCACTT3'
SF3a3	pGEX2T-PL	5' BamHI: 5'CGCGGATCCTTACCCAAAGAC3'
SF3a3	pGEX2T-PL	3' EcoRI: 5' CGGAATTCGAGAGTTCACTT3'
TFIIE-β	pGEX2T-PL	5' BamHI; 5' CCGGATCCTTACCCTCAGGCTCT3'
TFIIE-β	pGEX2T-PL	3' XhoI: 5' CCGCATAGATCTCTCGAGATTTACC3'
Radixin	pGEX2T-PL	5' BamHI: 5'CGGGATCCCCGGACGCTGAGCTA 3'
Radixin	pGEX2T-PL	3' XhoI: 5'GATCTCTCGAGATTTACCGCCC 3'

Oligonucleotides used to construct the integrase fusions in pSH2-1, pNlexA, pmalc2, the LEDGF clones in pSH2-1, pGADNOT, and pGEX2TPL. Oligos used to construct GST fusions of Ku70, PRC, SF3a3, TFIIE-β, and Radixin in pGEX2TPL.
